# Tapering of prescribed opioids in patients with long-term non-malignant pain (TOPIO)—efficacy and effects on pain, pain cognitions, and quality of life: a study protocol for a randomized controlled clinical trial with a 12-month follow-up

**DOI:** 10.1186/s13063-021-05449-5

**Published:** 2021-07-28

**Authors:** Grelz Henrik, Midlöv Patrik, Håkansson Anders, Jakobsson Ulf, Rivano Fischer Marcelo, Ringqvist Åsa

**Affiliations:** 1 Department of Neurosurgery and Pain Rehabilitation, Skåne University Hospital, Lasarettsgatan 13, 221 85 Lund, Sweden; 2 Center for Primary Health Care Research, Faculty of Medicine Department of Clinical Sciences Malmö, Lund University, Jan Waldenströms gata 35, 202 13 Malmö, Sweden; 3 Department of Clinical Sciences Lund, Faculty of Medicine, Psychiatry, Lund University, Baravägen 1, 221 00 Lund, Sweden

**Keywords:** Opioid, Long-term pain, Chronic pain, Prescribed, Tapering, Quality of life, Mental health, Physical health, Functioning, Opioid use disorder

## Abstract

**Background:**

Opioids are still widely prescribed to long-term pain patients although they are no longer recommended for long-term treatments due to poor evidence for long-term efficacy, risks of serious side effects, and the possibility of inducing opioid hyperalgesia. In a Cochrane study from 2017, the authors identified an urgent need for more randomized controlled trials investigating the efficiency and effects of opioid tapering.

The study aimed to assess (1) the efficiency of a structured intervention in causing stable reductions of opioid consumption in a population with long-term non-malignant pain and (2) effects on pain, pain cognitions, physical and mental health, quality of life, and functioning in response to opioid tapering.

**Methods:**

The study is a randomized controlled trial. The sample size was set to a total of 140 individuals after estimation of power and dropout. Participants will be recruited from a population with long-term non-malignant pain who will be randomly allocated to (1) the start of tapering immediately or (2) the control group who return to usual care and will commence tapering of opioids 4 months later. A 12-month follow-up is included.

When all follow-ups are closed, data from the Swedish drug register of the National Board of Health and Welfare will be collected and individual mean daily opioid dose in morphine equivalents will be calculated at three time points: baseline, 4 months, and 12 months after the start of the intervention. At the same time points, participants fill out the following questionnaires: Numeric Pain Rating Scale (NPRS), Tampa Scale of Kinesiophobia (TSK), Pain Catastrophizing Scale (PCS), Chronic Pain Acceptance Questionnaire (CPAQ-8), Hospital Anxiety and Depression Scale (HADS), and RAND-36. At baseline and follow-up, a clinical assessment of opioid use disorder is performed.

**Discussion:**

A better understanding of the efficiency and effects of opioid tapering could possibly facilitate attempts to taper opioid treatments, which might prove beneficial for both the individual and society.

**Trial registration:**

ClinicalTrials.gov NCT03485430. Retrospectively registered on 26 March 2018, first release date. “Tapering of Long-term Opioid Therapy in Chronic Pain Population. RCT with 12 Months Follow up (TOPIO).” First patient in trial 22 March 2018.

## Administrative information

**Table:** 

Title	Tapering of prescribed opioids in patients with long-term non-malignant pain (TOPIO) – Efficacy and effects on pain, pain cognitions, and quality of life: a study protocol for a randomized controlled clinical trial with a twelve-month follow-up	
Trial registration {2a and 2b}.	Registered in Clinicaltrials.gov with id: NCT03485430, “Tapering of Long-term Opioid Therapy in Chronic Pain Population. RCT with 12 Months Follow up (TOPIO)”. First release date: 26/03/2018, retrospectively registered. First patient in trial 22/03/2018. https://clinicaltrials.gov/ct2/show/NCT03485430.	
Protocol version {3}	Record log in Clinical Trials:	
	06/22/2021	Author’s comment: Collaborating Centers in Gothenburg and Linkoeping are added as intervention expands to a multi-center trial. These centers will be recruiting participants and performing intervention with start autumn 2021. As no inpatient tapering has been performed since trial start this way of conducting intervention is no longer permitted.
	09/01/2020	Author’s comment: Acronym added to title. Exclusion criteria more thoroughly described but has been the same since start in March 2018. The trial has only recruited patients with prescribed opioids and not recruited participants with illicit drug use.
	05/07/2020 09:16 ClinicalTrials.gov	PRS Review comments recorded. Listed each Outcome Measure separately, using “Add a __ Outcome Measure” feature, please use/keep this format for future records. ClinicalTrials.gov QA9
	05/06/2020 05:54	New power-calculation has been performed January 2020. Study period is prolonged due to this. Outcome measurements has been more thoroughly described in this last entry. Data-collection has been the same since start in March 2018.
	03/30/2018 14:18 ClinicalTrials.gov	Made minor editorial changes (e.g., spelling, grammar, punctuation). ClinicalTrials.gov QA37
Funding {4}c	Lund University Hospital, Lund University, Southern Healthcare Region and Greta and Johan Kock’s Foundation.	
Author details {5a}	Grelz Henrik: Department of Neurosurgery and Pain Rehabilitation, Lasarettsgatan 13, Skåne University Hospital, 221 85 Lund, Sweden and Lund University, Department of Clinical Sciences Malmö, Center for Primary Health Care Research, Jan Waldenströms gata 35, 202 13 Malmö, Sweden Midlöv Patrik: Lund University, Department of Clinical Sciences Malmö, Center for Primary Health Care Research, Jan Waldenströms gata 35, 202 13 Malmö, Sweden Håkansson Anders: Lund University, Department of Clinical Sciences Lund, Faculty of Medicine, Psychiatry, Sweden, Baravägen 1, 221 00 Lund, Sweden Jakobsson Ulf: Lund University, Department of Clinical Sciences Malmö, Center for Primary Health Care Research, Jan Waldenströms gata 35, 202 13 Malmö, Sweden Rivano Fischer Marcelo: Department of Neurosurgery and Pain Rehabilitation, Lasarettsgatan 13, Skåne University Hospital, 221 85 Lund, Sweden and Lund University, Department of Clinical Sciences Malmö, Center for Primary Health Care Research, Jan Waldenströms gata 35, 202 13 Malmö, Sweden Ringqvist Åsa: Department of Neurosurgery and Pain Rehabilitation, Lasarettsgatan 13, Skåne University Hospital, 221 85 Lund, Sweden and Lund University, Department of Clinical Sciences Malmö, Center for Primary Health Care Research, Jan Waldenströms gata 35, 202 13 Malmö, Sweden.	
Name and contact information for the trial sponsor {5b}	Primary sponsor: Department of Neurosurgery and Pain Rehabilitation, Lasarettsgatan 13, Skåne University Hospital, 221 85 Lund, Sweden. Contact persons: Grelz Henrik, MD +4646172289 e-mail: henrik.grelz@med.lu.se Sponsor’s reference: RS-id 130656 Åsa Ringqvist, MD PhD e-mail: Asa.Ringqvist@skane.se Secondary sponsor: Lund University, Department of Clinical Sciences Lund, Faculty of Medicine, Psychiatry, Sweden, Baravägen 1, 221 00 Lund, Sweden Principal Investigator and Contact person: Midlöv, Patrik e-mail: patrik.midlov@med.lu.se	
Role of sponsor {5c}	Lund University Hospital is the trial sponsor with the main collaborator Lund University. No one outside the group of researchers will be involved in the data collection, analysis, interpretation or writing the present manuscript. .	

## Introduction

### Background and rationale {6a}

In 1986, a study including 38 patients with long-term non-malignant pain concluded that opioids could be prescribed safely on a long-term basis [1]. Despite low-quality evidence, the paper was widely cited and greatly influenced prescription patterns for patients with long-term pain. Opioid use increased gradually, and in 1996, the rate of opioid use began accelerating rapidly after the introduction of oxycodone [2, 3]. An opioid epidemic has later been described in the USA caused by rises in opioid prescription use but also social determinants, e.g., lack of education [4]. It imposes a major threat to public health with a dramatic threefold increase in opioid overdose deaths from 1999 to 2017 [5].

Further studies have not been able to establish advantages of opioid treatments exceeding 3 months for long-term pain but rather pointing out disadvantages with long-term opioid therapy [6, 7]. A Cochrane review of opioids for pain associated with rheumatoid arthritis found limited evidence for the efficacy of weak opioids up to 6 weeks and no evidence beyond 6 weeks [8]. In lumbago, evidence indicates a short-term effect on pain if the treatment continued for less than 8 weeks though not proven more efficacious than NSAID. Opioid treatment was, however, associated with worse prognosis for return to work [9]. In this context, it is interesting to note publicly available information from the Swedish National Board of Health and Welfare displaying prescription patterns of individuals applying for sick leave. In this report, 42% of patients with the diagnosis of lumbago were prescribed opioids before sick leave with an increase to 70% after the initiation of sick leave [10]. In Sweden, opioids do not, however, constitute a problem of equal magnitude as in the USA. Information made accessible by the Swedish National Board of Health and Welfare indicates a stable prescription rate with a tendency toward a slight decrease in the number of individuals receiving opioid dispensation from 10.2% in 2006 to 9.38% in 2015 [11].

Thus, increasingly emphasized by clinical guidelines and strategic document, long-term opioid treatments are not considered evidence-based practice [6, 12, 13]. The International Association for the Study of Pain (IASP) has in a statement 2018 recommended caution when prescribing opioids for long-term pain and concluded that preferred treatment strategies are interventions that aim at improving the quality of life, especially those integrating behavioral and physical treatments [14]. Besides not been proven efficacious in the treatment of long-term pain, opioid treatments can also lead to serious side effects such as addiction, overdose, and death by overdose. Less dramatic but more frequent side effects are constipation, nausea, sedation, and increased risk of falls and fractures. Depression and sexual dysfunction may also develop over time. In addition, cognitive side effects are prevalent in populations receiving long-term opioid therapy [6, 7, 15, 16].

Side effects of long-term opioid medication can thus hamper both physical and mental health as well as function. Moreover, the phenomenon opioid-induced hyperalgesia has generated increasing attention [17–20]. Opioid-induced hyperalgesia is defined as a heightened state of nociceptive sensitization instigated by the exposure of opioids, which leads to the paradoxical effect whereby a patient receiving opioids for the treatment of pain can become more sensitive to painful stimuli. Opioid-induced hyperalgesia could thus possibly explain the loss of opioid efficacy in some patients.

To sum up, there are good reasons to be careful when prescribing opioids for long-term pain due to poor evidence for long-term efficacy, risks for serious side effects, addiction, negative impact on return to work, and, finally, the possibility of inducing hyperalgesia by the very exposure of opioids. Nevertheless, it can be challenging to help patients taper and end their opioid treatment. Both clinicians and patients may struggle with beliefs that might hamper the process. Qualitative data points to challenges in patients’ beliefs such as the fear of increased pain with opioid tapering [21, 22]. Clinicians, on the other hand, predict negative reactions from patients upon a recommendation to reduce opioid intake, varying from resistance to change to becoming overtly angry. Consultations entailing discussion of opioid tapering are by some clinicians described as emotionally charged, exhausting, and even threatening [23, 24]. Both patients’ and clinicians’ beliefs might thus constitute barriers for successful change in opioid prescriptions. The communication about tapering is facilitated if patients understand their individualized reasons for tapering, are encouraged to have input into the process, and are assured they will not be abandoned [25]. Patients with opioid tapering experience identified social support and a trusted health care provider as facilitating factors for their process [21]. There are guidelines on how to taper opioids with practical advice where tapering of the long-term treatment is described as a method to determine the benefit–harm ratio for the individual patient [26, 27]. An improved research-based understanding on what patients can expect when entering an opioid tapering program could very possibly be helpful in this demanding clinical situation.

A Cochrane systematic review conducted in 2017 attempted to investigate the effectiveness of different methods to taper prescribed opioids [28]. The authors concluded that there was no evidence for the efficacy or safety of methods for reducing prescribed opioid use in long-term pain. They found only five randomized controlled trials (RCTs), the designs were too heterogeneous to allow for a meta-analysis, and the findings were mixed. The authors saw a vital need for more RCTs that focus on opioid medication use in patients with long-term pain with reduction of opioid dose as a primary clinical goal together with measures of patient-reported outcomes of pain, mood, and functioning. The present RCT is thus designed to meet these requirements in order to be beneficial for patients, clinicians, and researchers.

### Objectives {7}

Primary objective: To determine if tapering of medically prescribed opioids in a long-term non-cancer pain population is superior to control in reducing dispensed opioids and non-inferior regarding participants’ report on pain, pain cognitions, physical and mental health, quality of life, and functioning.

Secondary objective: To determine if a reduction in opioid intake displays stability over time.

Research hypothesis: Tapering of opioids in a structured intervention will reduce opioid intake by at least 50% and is superior to controls in reducing dispensed opioids.

### Trial design {8}

**Fig. 1:**
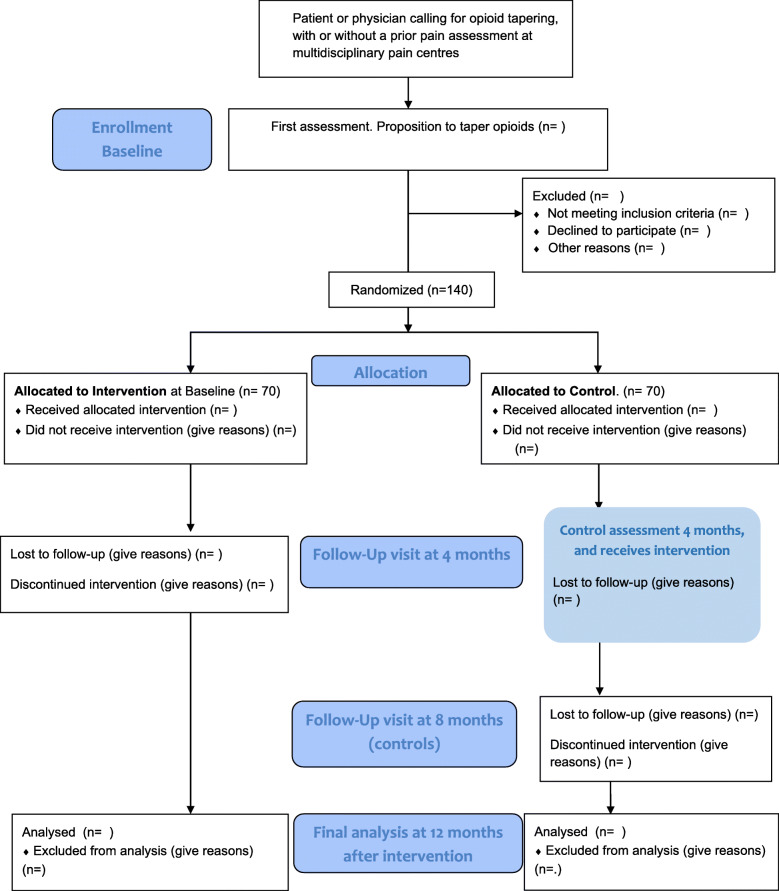
Flow-chart TOPIO from baseline to follow-up

The design of the present study is an RCT, which is under recruitment and conducted among patients with long-term pain who have been accepted to undergo a structured intervention aiming to taper opioid long-term treatments. The study population will be randomly allocated to either (1) the start of tapering opioids immediately or (2) the control group who will return to usual care and commence tapering of opioids 4 months later. A 12-month follow-up after the start of the intervention is included (Fig. 1). The study lies within a superiority framework. Primary endpoints are hypothesized to generate a decrease in opioid use in comparison to controls.

## Methods: participants, interventions, and outcomes

### Study setting {9}

Participants will be recruited from a population referred to tertiary pain clinics at three sites in Sweden: (1) Pain Rehabilitation Clinic of Skane University Hospital in Lund, (2) Pain Centre Sahlgrenska University Hospital in Gothenburg, and (3) Pain and Rehabilitation Centre in Linköping.

### Eligibility criteria {10}

Inclusion criteria are age ≥ 18 years with long-term (> 3 months) non-malignant pain and long-term prescribed opioid consumption (> 3 months) with a participant and/or prescribing doctor calling for tapering of opioids. All pain classes will be included, i.e., nociceptive pain (both somatic (musculoskeletal) and visceral), neuropathic pain, and nociplastic pain. Tapering of opioids is thus proposed without regard to pain mechanism or diagnoses. The requirement regarding functioning is (1) that a participant is able to absorb and retain information and is thus able to make an informed decision and (2) is at a functional level that permits outpatient visits.

Exclusion criteria at baseline are repeated intake of non-prescribed opioids or other illegal drugs, refusal to perform drug screening, prescription periods shorter than 3 months, and perceived medical risks of waiting with tapering of opioids (e.g., raising opioid doses after acute pancreatitis caused by excessive alcohol intake or kidney disease stage 4–5 in combination with excessive alcohol intake and high doses of opioids). The occurrence of substance use disorder without the use of illicit drugs is not an exclusion criterion.

Exclusion criteria were selected as a safety option to the physician at baseline visit when estimating the individual risks of the ongoing opioid treatment with regard to both overdose and development of dependence whenever a short duration (< 3 months) of treatment has occurred.

Otherwise, eligible participants who chose to not be included in the Swedish Quality Registry for Pain Rehabilitation (SQRP) will also be excluded.

The intervention will be carried out by physicians and nurses employed at tertiary pain clinics.

### Who will take informed consent? {26a}

Prior to baseline visits, written information is mailed to participants and, when scheduling an appointment, oral information is also provided by a study nurse. At baseline visits, the trial is again described orally, and informed consent is obtained by physicians.

### Additional consent provisions for collection and use of participant data and biological specimens {26b}

Not applicable.

## Interventions

### Explanation for the choice of comparators {6b}

It was considered non-ethical to deny patients interested in tapering of opioids the intervention. The study was therefore designed to use waiting list as the control group with a cross-over design receiving intervention after 4 months.

### Information to and screening of all participants before randomization

Written and oral information before baseline visit is provided to each eligible participant on:

- The study design and the randomization procedure
- The rationale for the study and information on dosages that might cause harm and possible symptoms at withdrawal
- The possibility to withdraw consent to participate at any time point with no requirement to motivate the decision

At baseline visits, additional information is provided and screening is carried out.

Topics that are covered during the baseline visit:

- The participant’s understanding of his/her long-term non-cancer pain is discussed, and when called for, information is provided.
- A structured screening of possible opioid-related side effects that the patient might experience is performed.
- Possible side effects of long-term opioid therapy at the group level as well as reported by the participant are discussed in connection to the patient’s conceptualization of the advantages of the opioid treatment.
- Evidence-based pharmacological treatments for long-term pain, i.e., serotonin–norepinephrine reuptake inhibitor (SNRI), tricyclic antidepressants, and gabapentinoids.
- The need for study subjects to, during the trial, give the prescribing physician access to the national register of prescribed medications to follow dispensing of opioids.
- The participant’s daily intake of opioid converted to milligram morphine equivalent per day (MME) is calculated. If the consumed median daily opioid dose in morphine equivalents exceeds 100 mg, the patient is informed of the increased risks of dangerous overdose and tapering is thus strongly suggested. If benzodiazepines are co-prescribed, tapering of opioids is also strongly suggested.
- The tapering procedure and possible symptoms of withdrawal that might appear during tapering are described.

#### Screening

The initial part of the evaluation process encompasses a structured interview using DSM-5 substance use disorder criteria, urine drug screening, and blood sampling and analyses of Phosphatidylethanol 16:0/18:1 (B-PEth). Drug screening is performed for detection of morphine, codeine, heroin, oxycodone, buprenorphine, methadone, tramadol, benzodiazepines, cannabinoids, amphetamine, and cocaine with confirmatory testing on the accredited laboratory when unexpected findings occur. A blood test for B-PEth is made to detect levels of alcohol use during approximately the last 2 weeks. Refusal to perform the tests is evaluated as equal to a positive test. Interview, laboratory testing, and clinical judgment are all considered to evaluate the presence of SUD according to DSM-5 criteria [29].

### Intervention description {11a}

After randomization and at the baseline visit, a written individual treatment plan is established by the physician and reviewed by the nurse and participant in concordance with guidelines [27, 30].

The written treatment plan includes the following:

- Information on well-known risks and side effects of long-term opioid treatments of long-term pain.
- Possible symptoms of withdrawal that might appear during tapering.
- The participant’s daily intake of opioid converted to milligram morphine equivalent dose per day (MME).
- The participants’ individual goals with tapering opioids.
- Information about follow-up: weekly follow-up at the commencement of tapering. Nurses’ follow-up is then performed according to participants’ needs which can be weekly or less frequent.
- The architecture of the tapering process where the participant is advised to decrease the dosage with approximately 5–10% from starting dose every week.
- The rationale for switching to a single preparation of extended-release opioid preparation if immediate-release opioids are used. If more than one type of opioid is used, tapering is performed with one drug at the time. If the patient is willing to taper pregabalin or benzodiazepines, this may be performed as well.
- Participant’s approval to prescribing doctor during the trial to use the Swedish drug register of the National Board of Health and Welfare. Opioids are prescribed with 7–30-day intervals or may be delivered by multi-dose dispensing by staff in intervals of 1 or 2 weeks if there are problems in following prescriptions.
- Information about urine drug screening and blood test for B-PEth that may occur at any time during the trial and should be performed on demand within 48 h. Refusal to perform the test is equal to a positive test. Results on drug screening are delivered approximately 1 week after sampling and are sent by post. If drug screening at baseline is without remark, no more drug screening is performed during tapering.
- Consequences that may occur if the treatment plan is not followed.
- The collaborative decision physician–participant on however evidence-based and safe pharmacological treatments for long-term pain, i.e., serotonin–norepinephrine reuptake inhibitor (SNRI), amitriptyline, and gabapentin, is in question to be tested after or during tapering.
- During tapering, rescue medication may be used. NSAID used for increased pain is and alimemazine, mirtazapine, or equal drug is in question to use for sleeping disturbance.
- Named contact nurse during the trial.

Follow-up will be performed by a nurse, preferably by phone, but can be made possible in person. Visits are necessary if multi-dose drug dispensing proves necessary. The number of contacts with the nurse is optional and decided by the participant in cooperation with the nurse. During tapering, the nurse may slow down tapering if participants are having problems following the plan or report a considerable decline in health and function.

#### Follow-up visit at 4 months

Four months after initiation, a visit with a study physician and nurse is performed:

- Obstacles and successes are discussed. Personal experiences and conclusions are important topics of this meeting.
- In case of the remaining daily intake of opioids and if the participant is interested in further support in tapering, this demand is met if considered possible within a well-defined timeframe.
- Assessment of non-narcotic medications for the treatment of long-term pain including dosage and suggested treatment period.
- Assessment of further needs for the participant, e.g., whether participation in interdisciplinary multi-modal pain rehabilitation in a group is called for or other individual approaches of rehabilitation at the primary health care level.
- Clinical assessment as to whether any interventions from opioid disorder care are warranted.
- Drug screening including B-PEth is performed.
- The visit and outcome of the intervention complete with recommendations regarding future prescription are summarized and communicated to the physician. The recommendations are created in collaboration with the participant.

### Criteria for discontinuing or modifying allocated interventions {11b}

Non-compliance to the treatment plan or drug screening showing evidence of a drug not prescribed calls for attention from a physician. Additional screening for a multiple of drugs available on the illicit market may be performed. Renewal of the treatment plan is offered, and the participant may be retained in the study and final analysis.

If the treatment plan, despite this, is not followed, the participant is referred to an addiction care center and excluded from the final analysis.

There are no restrictions due to concomitant care and interventions during the trial.

### Strategies to improve adherence to interventions {11c}

A motivational approach is used in the consultation at the start of tapering. Participant’s worries, thoughts, and expectations are explored. Firstly, the patient’s perspective of opioid use is considered. Time is spent on reflections and concerns relating to the tapering process. Increased pain during tapering is a well-known fear. The patient’s history and experience of potential side effects are linked with known risks of opioids in an individualized manner, and the patient is reassured of the possibility to contact nursing staff on office hours.

Compliance will be monitored mainly by telephone support by an assigned study nurse once a week in the beginning of tapering. It is optional to attend in person. Distribution of opioids once a week may occur by decision from the physician or if preferred. Monitoring of symptoms related to abstinence or other symptoms is the main target as well as monitoring the willingness to continue the tapering plan. Participants will also be advised to take contact if problematic symptoms prevail.

Once a week, physicians and nurses meet to discuss obstacles in tapering. The main strategy to deal with increased symptoms is to change the speed of tapering and make adjustments with non-narcotic drugs (i.e., NSAID, paracetamol, alimemazine, amitriptyline, duloxetine, gabapentin).

### Relevant concomitant care permitted or prohibited during the trial {11d}

Participants cannot participate in interdisciplinary pain rehabilitation programs during the tapering period. Unimodal treatment as in usual care is however permitted.

### Provisions for post-trial care {30}

All participants are covered by the national health insurance system in the event of suffering harm during the trial.

### Outcomes {12}

Outcome measurements are chosen in regard to recommendations for clinical trials in a chronic non-cancer pain population [31]. Primary outcomes are chosen as evaluation of methods on tapering trials in line with recommendations [28].

#### Primary outcomes

Consumption of opioids, measured as MME, will be evaluated using data extracted from the Swedish drug register of the National Board of Health and Welfare (SDPR) where registration of all withdrawals of prescribed drugs is made. MME will be calculated using these data. At all baseline visits and 12-month follow-ups, the MME will be calculated for the preceding 90 days, but at 4 months after the intervention, the dose will be calculated for the preceding 30 days as the intervention is estimated to last approximately 3 months.

Self-reported opioid consumption will also be evaluated and reported in MME. Calculation of morphine equivalent dose will be performed with conversion factors used in Cochrane review [7].

#### Secondary outcomes

Secondary outcomes are effects on pain, pain cognitions, mental health, life quality, and physical and psychological functioning using validated questionnaires registered in SQRP [32]. Pain rating will be evaluated using NPRS. Pain cognitions are mapped with the Tampa Scale of Kinesiophobia (TSK), Pain Catastrophizing Scale (PCS), and Chronic Pain Acceptance Questionnaire (CPAQ-8). Hospital Anxiety and Depression is applied to assess mental health, and RAND-36 will be used to assess the quality of life and functioning entailing physical, mental, and general health.

##### Numeric Pain Rating Scale (NPRS)

NPRS was used to capture the patient’s level of pain intensity. Patients rate their average level of pain during the last week. The 11-point scale spans from the left with the phrase “no pain,” i.e., 0, and on the right to the phrase “worst imaginable pain,” i.e., 10. Numeric pain scales have been shown to be reliable and valid [33–35].

##### Pain in anatomical regions

Participants report pain besides intensity also from different anatomical regions in the body including the head, joint, and soft tissue. There are 36 sites optional for the report. The number of pain sites will be analyzed.

##### Tampa Scale of Kinesiophobia (TSK)

The fear-avoidance model applied on long-term pain led up to the introduction of the term “kinesiophobia” and the development of TSK [36, 37]. Kinesiophobia was defined as “a condition in which a patient has an excessive, irrational and debilitating fear of physical movement and activity resulting from a feeling of vulnerability to painful injury or re-injury.” TSK has been found to strongly predict levels of pain intensity and disability in patients with musculoskeletal pain [38].

The TSK employs a 4-point Likert scale, with scoring options ranging from 1 (strongly disagree) to 4 (strongly agree), and encompasses 17 items related to pain, fear of movement, and re-injury. The total score of the original 17-item version ranges between 17 and 68, with a higher score indicating a higher degree of kinesiophobia. The Swedish version has been tested and validated [39–41].

##### Pain Catastrophizing Scale (PCS)

The PCS comprises 13 items that are rated from 0 to 4 with the endpoints 0 (“Never”) to 4 (“All the time”) and was constructed to assess pain-related catastrophizing [42]. Catastrophizing includes three factors: (a) Helplessness, i.e., perceived helplessness in situations when pain is present (six items); (b) Rumination, concerning vigilance toward the pain experience (four items); and (c) Magnification, i.e., the tendency to magnify the threat value of pain (three items). The Swedish version has recently been validated with psychometric properties supporting internal consistency (Cronbach’s *α* 0.92) and structural validity as well as indicating support for the three-factor solution [43].

##### Chronic Pain Acceptance Questionnaire (CPAQ-8)

Pain acceptance measures two main classes of behaviors represented by respective subscales: Activity Engagement (“AE,” score range 0–24) and Pain Willingness (“PW,” inverted score range 0–24). The items are rated from 0 (never true) to 6 (always true) and higher values indicate higher acceptance to long-term pain. CPAQ-8 scales exhibit good internal consistency (alpha ≥ 0.80), correlate significantly with related constructs, and have also been tested for validity in people living with long-term pain [44, 45].

##### Hospital Anxiety and Depression Scale (HADS)

HADS was constructed for patients in medical settings [46]. It entails 14-item measures of anxiety (7 items) and depression (7 items) symptoms over the course of a week. Items are rated on a 4-point scale (0 = not all; 3 = very often) and the anxiety and depression subscales range from 0 to 21. Higher scores indicate greater severity. The cutoff points for anxiety and depression are 0–7 for non-cases, 8–10 for doubtful cases, and 11–21 for cases. The Swedish version applied in this study has exhibited excellent internal consistency for the total (*α* = 0.90), anxiety (*α* = 0.84), and depression scales (*α* = 0.82) and has correlated significantly with other measures of anxiety and depression consistent with the English original [47].

##### RAND-36

The non-profit organization RAND Corporation USA, promoting Medical Outcomes Study, originally developed SF-36. The Swedish RAND-36 is a modern translation of SF-36. Recent psychometric evaluation of the Swedish version gave support for the reliability and responsiveness [48]. RAND-36 reflects current perceptions of health. Subjects are asked questions related to how well they have been coping during the last 4 weeks.

RAND-36 encompasses questions from eight domains (subscales), i.e., physical functioning, role physical, bodily pain, general health, vitality, social functioning, role emotional, and mental health. The scores are subsequently transformed in a standardized way into a 0–100 scale where higher scores indicate better health. Minor differences exist between RAND-36 and SF-36 in scoring procedures for bodily pain and general health of the eight subscales. The RAND-36 does not have an authorized algorithm for calculating Mental and Physical Component Summary scores.

##### Use of health care

Reports of the utilization of health care due to long-term pain during the last 12 months are collected. “How many times have you sought medical assistance by a physician due to your pain in the last twelve months” with the following options: 0–1, 2–3, or more than 4 times. Patients reporting more than four visits in the last 12 months tend to have a multitude of visits [49, 50].

##### Baseline characteristics

Characteristics including age, gender, length, weight, country of birth, education, and employment/sickness compensation are included in baseline questionnaires.

#### Additional data collection

##### Presence of substance use disorder (SUD)

A non-validated structured interview is performed based on DSM-5 criteria for SUD and information about the clinical evaluation for the presence of SUD [29]. Information regarding the presence of SUD in the family is also collected in this interview.

##### Drug screening

Analysis of prescribed and illicit drugs is performed in the first line by enzyme-immune analysis.

If the test results indicate the use of a drug that is not prescribed, a mass spectrometry method is used.

##### Opioid-induced symptoms

A non-validated instrument for the measurement of self-reported symptoms during opioid use will be used at baseline and at the 4-month follow-up. It employs a 5-point Likert scale from 0 (no symptoms) to 5 (severe symptoms) where higher points indicate more symptoms. The following symptoms will be monitored: difficulties to concentrate, mood disturbance (anxiety or depression), constipation, diarrhea, nausea, vomiting, anorexia, dry mouth, fatigue or easily worn out, sleepy or fall asleep easily in daytime, dizziness, headache, flushing, sweating, and itching. Other bothersome symptoms can also be reported by the participant. This scoring is used in the trial in the clinical evaluation of tapering effects experienced by the participants.

##### Global opinion about tapering

At follow-up, all participants are asked: How do you consider your life situation has been affected by tapering opioids? Possible answers are (1) improved, (2) no change, and (3) impaired.

##### Opioid replacement therapy

Opioid replacement therapy is not registered in the SPDR as buprenorphine or methadone is dispensed at addiction care centers. Data is therefore derived from medical records at 12 months after the received intervention.

### Participant timeline {13}

Participants will be enrolled in the trial for 12 months (intervention) or 16 months (control) following baseline assessment. The structured tapering intervention has a 4-month duration, but may be prolonged.

Participants denying tapering, but accepting to participate, will be followed up after 12 months for dropout analysis (Fig. 1).

A SPIRIT diagram (Table 1) is provided detailing the timing of enrolment, interventions, and assessments. Any methodological changes in the study design or sample size that may potentially affect the participants’ safety or study procedures will be discussed in the committee of ethics.

**Table 1:**
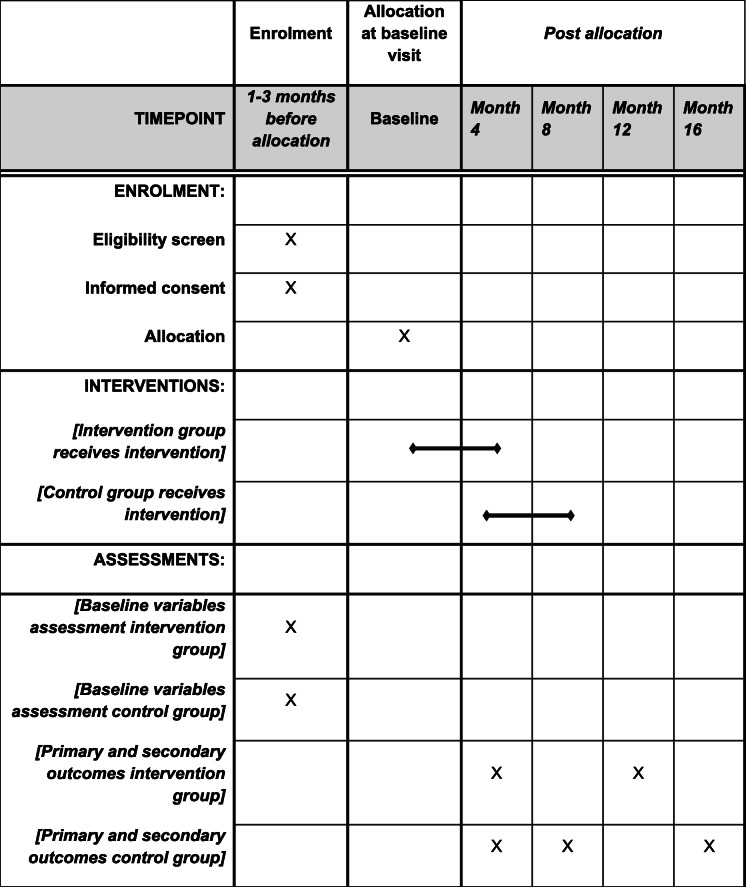
SPIRIT diagram

Schedule of enrolment, interventions, and assessments

### Sample size {14}

A “responder” to treatment is a participant who experiences at least a 50% reduction in opioid consumption [28]. Estimation of sample size was based on an effect size of 0.5 (Cohen’s *d*) 80% power, *α* = 0.05, and drop-off at 10%. The results from the calculation indicated the need for 70 individuals in each group.

### Recruitment {15}

To reach the sample size, additional sites at pain clinics in other parts in Sweden were invited during 2020/2021.

## Assignment of interventions: allocation

### Sequence generation {16a}

The allocation sequence is generated by computer to intervention or control in ratio 1:1 and is thereafter kept in sealed envelopes prepared by independent staff for an allocated group of 140 participants. Sealed envelopes will be kept in Lund and allocation will consequently take place in Lund.

### Concealment mechanism {16b}

Blinding of participants or health care professionals is not possible due to the nature of the intervention, and no blinding will be performed in the assessment of outcomes or data analysis.

### Implementation {16c}

The sealed envelopes are stored in a locked storage and will be assigned and opened by a physician at baseline visit upon a decision of the participant to participate in a structured tapering process.

## Assignment of interventions: blinding

### Who will be blinded {17a}

No blinding at any time will be performed.

### Procedure for unblinding if needed {17b}

Not applicable.

## Data collection and management

### Plans for assessment and collection of outcomes {18a}

#### Primary outcomes

Individual data from the SDPR will be collected when all follow-ups are closed [51]. Self-report of daily opioid consumption at the time of the visit will also be assessed.

#### Secondary outcomes

Before a baseline visit, a phone call by a study nurse to orally inform about the trial is made and a questionnaire with secondary outcomes and baseline characteristics is mailed to participants as a part of reporting to SQRP, but with a reduced content for the study population [32]. Participants denying tapering are reported to SQRP only at baseline visits. A fulfilled questionnaire is required to be scheduled at a baseline visit for all participants. The baseline visit must occur within 3 months after the questionnaire has been filled out.

The follow-up questionnaire is sent by mail 1 month before time points: 1 month (all participants), 8 months (controls), and 12 months (all) after the start of tapering.

Twelve months after the start of tapering, a phone call by administration staff is followed by a mailed questionnaire.

### Plans to promote participant retention and complete follow-up {18b}

#### Primary outcomes

MME will be retrieved from SDPR for participants who discontinue or deviate from the protocol.

#### Secondary outcomes

If the questionnaire is not returned within 3 weeks after being mailed, a telephone reminder will be performed. The follow-up visit will be scheduled at 4 and 8 months regardless of participants returning the SQRP questionnaire before the visit or not. If not completed before the visit, the participant will be given the opportunity to fill out the questionnaire at the time of the visit.

At 12 months, a telephone reminder will be performed if the questionnaire is not returned within 4 weeks. Hereafter, no further attempt is made to contact those who do not respond.

### Data management {19}

*Primary outcomes* are derived from the SDPR where all dispensed prescribed drugs are registered. The reliability of prescribed drugs at an individual level is good. Statistics are based on statutory reporting from pharmacies. Data collection is, to a vast extent automated, where data are extracted from administrative systems [51].

*Secondary outcome* data entry into SQRP is performed by medical staff according to standard clinical procedures at the department as they are part of the standard evaluation. A case report form is designed by SQRP’s Clinical Data Management team and it is used by all units reporting to SQRP. To secure validity, a tenth of the entry will be randomly checked. In case of discrepancies, all entries will be checked.

*Additional data* are transferred into electronic data capturing system, REDCap®, in accordance with a written case report form regarding information about self-report of opioid consumption the last week, allocation, date of written agreement, dates for visits, dates for follow-ups, frequency of contacts with the nurse, opioid-induced symptoms, structured interview of DSM-5 substance use disorder, drug screening, clinical assessment of substance use disorder, drop-offs, adverse events, global opinion about tapering effects on health, and review of medical records regarding opioid replacement therapy at 12 months.

### Confidentiality {27}

Data confidentiality will be protected in accordance with the routines of SDPR and SQRP. Additional data kept in REDCap® will be coded with study ID at each study center, and no other data providing personal identification will be kept. Linking of databases will be performed by the National Board of Health and Welfare.

### Plans for collection, laboratory evaluation, and storage of biological specimens for genetic or molecular analysis in this trial/future use {33}

Not applicable.

## Statistical methods

### Statistical methods for primary and secondary outcomes {20a}

All statistical analyses will be performed with the SPSS software (version 25, IBM Inc). Continuous data will be expressed by means, standard deviation and 95% confidence interval, and categorical data in numbers and percentages.

The intervention arm (tapering opioids) will be compared with control (waiting list) for primary and secondary outcomes at 4 months of follow-up. Statistical analysis of differences between groups will be performed per protocol (attending follow-up 4 months after the start of the intervention and attending second baseline visit in controls) with a paired *t*-test regarding the primary outcome and a Wilcoxon signed-rank test regarding secondary outcomes.

### Interim analyses {21b}

There will be no interim analyses.

### Methods for additional analyses (e.g., subgroup analyses) {20b}

#### Retention to intervention at 12 months

Analysis of retention to the tapered dose will be performed with a paired analysis within the allocated group. Changes in percent from the time point for follow-up after the received intervention at 4 months (intervention) or 8 months (control) will be calculated. The analysis will be performed with repeated measures ANOVA.

### Methods in analysis to handle protocol non-adherence and any statistical methods to handle missing data {20c}

When calculating total scores of TSK, PCS, CPAQ-8, HADS, and RAND-36, missing values may occur. These will be managed in accordance with guidelines from SQRP’s Clinical Data Management team. These are:

- TSK: two missing values are replaced by, at the individual level, the mean score for all replies of the instrument. More than two missing values are not allowed.
- PCS: two missing values are replaced by, at the individual level, the mean score for all replies of the instrument. More than two missing values are not allowed.
- CPAQ-8: no missing values are allowed.
- HADS: two missing values are allowed in each subscale anxiety and depression. Two missing values for each subscale are replaced by, at the individual level, the mean score for all replies of the subscale. More than two missing values per subscale are not allowed.
- RAND-36: missing values in each subscale are considered. Half the reported questions + one must occur if a total score is to be reported from a subscale. Missing values are replaced by the mean value of the remaining scores in the current subscale.

### Plans to give access to the full protocol, participant-level data, and statistical code {31c}

The data that supports the findings of this study are available from SQPR and SDPR, but restrictions apply to the availability of these data, which were used under license for the current study, and so are not publicly available. Data are, however, available from the authors upon reasonable request and with permission of SQPR and the Swedish drug register of the National Board of Health and Welfare. There is no plan to grant public access to the final dataset.

All authors will have access to the final coded trial dataset.

## Oversight and monitoring

### Composition of the coordinating center and trial steering committee {5d}

Pain Rehabilitation Clinic of Skane University Hospital in Lund will coordinate the trial. Organizational support to Pain Centre Sahlgrenska University Hospital in Gothenburg and Pain and Rehabilitation Centre in Linköping will be provided. Meetings between centers will take place once every second week to monitor the recruitment of participants, implementation of the intervention, and any problems in data collection [52].

### Composition of the data monitoring committee, its role, and reporting structure {21a}

No data monitoring committee is needed as the trial is not considered a drug trial.

Capturing of primary and secondary outcomes already has routines of SDPR and SQRP.

### Adverse event reporting and harms {22}

Adverse events are recorded and addressed (see also the “Strategies to improve adherence to interventions {11c}” section) for all participants during the trial and will be documented. Discontinuation might be called upon due to the need for specialized addiction care.

### Frequency and plans for auditing trial conduct {23}

No auditing is planned.

### Plans for communicating important protocol amendments to relevant parties (e.g., trial participants, ethical committees) {25}

Any modifications to the protocol which may impact on the conduct of the study and the potential benefit of the patient or may affect patient safety, including changes of study objectives, study design, patient population, sample sizes, study procedures, or significant administrative aspects, will require a formal amendment to the protocol. Such amendment will be approved by the Ethics Committee and changes in trial registration at ClinicalTrials.gov will be performed.

## Dissemination plans {31a}

All authors agree to disseminate the results from TOPIO regardless of its results. Dissemination will be through journal publication.

## Discussion

As described in the “Introduction” section, the problem of long-term opioid prescription periods and health care professionals feeling lost when it comes to helping their patients to discontinue treatments is significant. The present study could possibly give insights into the efficiency and effects of opioid tapering that could aid health professionals and patients and possibly facilitate attempts to taper opioid treatments. In order to have an impact, i.e., to facilitate dissemination, we wanted to perform this study as a well-described but straightforward physician–nurse collaboration without adding any specific psychotherapeutic techniques. In our opinion, this is crucial for this particular branch of health care to improve with possible benefits for both individuals and society such as reduced risks of developing substance use disorders and fatal overdoses.

## Trial status

The first participant was recruited in March 2018. To date, 62 patients are allocated to the intervention or control group. At this rate, the allocation process is assumed to proceed until 2024 to reach the goal of 140 randomized participants. With additional trial sites being added in 2021 and 2022, the recruiting process may end earlier. During the 2025 follow-up, data processing and manuscript preparation are planned.

The current version of the protocol: Clinical Trials.gov PRS 06/22/2021 by HGrelz, not yet publicly released.

Due to the COVID-19 pandemic, the recruitment process was brought to a halt in March 2020 and resumed on 1 September 2020 without any changes of the study population, but with an option for participants to take part in follow-up after intervention fully by phone or video. During January 2021, a halt in recruitment again took place for 1 month. Due to the COVID-19 pandemic, follow-up visits have been performed mostly by phone. Elderly, beyond the age of 70, was not allowed to enter into the trial between 7 January and 7 June 2021.

## Data Availability

Lund University Hospital is the trial sponsor with the main collaborator Lund University. No one outside the group of researchers will be involved in the data collection, analysis, interpretation, or writing the present manuscript. All authors will have access to the final coded trial dataset.

## Abbreviations

CPAQ-8: Chronic Pain Acceptance Questionnaire
HADS: Hospital Anxiety and Depression Scale
MME: Milligram morphine equivalent dose per day
NPRS: Numeric Pain Rating Scale
PCS: Pain Catastrophizing Scale
PEth: Phosphatidylethanol 16:0/18:1
RCT: Randomized controlled trial
SPDR: Swedish Prescribed Drug Register
SQRP: Swedish Quality Registry for Pain Rehabilitation
SUD: Substance use disorder
TSK: Tampa Scale of Kinesiophobia
